# Supplementary material for: Factors influencing scar formation following Bacille Calmette-Guérin (BCG) vaccination
Source: Heliyon. 2023 Apr 26;9(6):e15821. doi: 10.1016/j.heliyon.2023.e15821 (PMC10360588; doi:10.1016/j.heliyon.2023.e15821)
Supplement: Multimedia component 4 [file mmc4.pdf]

Supplemental Material 3

**What do you think about your scar ?**	n =2341
I don't mind having the scar at all	1806 (77%)
I would rather not have a scar, but understand this is unavoidable	472 (20%)
I'm dissatisfied with the scar	59 (3%)
*Unknown**	4 (<1%)

*4 participants with missing data

**Why are you dissatisfied with the scar?**	n =59
I didn't expect to have a scar	10 (17%)
It is worse than I expected	46 (78%)
*Other**	3 (5%)

*scar location, odd appearance, different appearance (purple/red) to that expected.

**Do you regret having the vaccine because of the scar?**	Total n =2341*	Australia n =1003*	Brazil n =1032*	Netherlands n =187	Spain n =52	UK n =67
No, I don't regret having the vaccine	2242 (96%)	931 (93%)	1023 (99%)	173 (93%)	51 (98%)	64 (96%)
Yes, I regret having the vaccine	88 (4%)	66 (7%)	4 (<1%)	14 (7%)	1 (2%)	3 (4%)

*11 participants with missing data (6 in Australia, 5 in Brazil)
