# Supplementary material for: Factors influencing scar formation following Bacille Calmette-Guérin (BCG) vaccination
Source: Heliyon. 2023 Apr 26;9(6):e15821. doi: 10.1016/j.heliyon.2023.e15821 (PMC10360588; doi:10.1016/j.heliyon.2023.e15821)
Supplement: Multimedia component 5 [file mmc5.pdf]

**Supplemental Table 1.** Sensitivity analysis; factors investigated for association with BCG scar formation (Australia)
Abbreviations: BCG, Bacille Calmette-Guérin; BMI, body mass index; LTBI, latent tuberculosis infection; NA, not applicable; OR, odds ratio; TST, tuberculin skin test
*Wheal response (yes/no) analysed for participants who received one BCG dose only.
Significant factors (p-value < 0.2) resulting from the univariate logistic regression analysis were included as possible covariates in a multivariate logistic regression model. The model presented in the table was created using backward stepwise exclusion of factors with p-value > 0.05, using sequential model testing.

| Factor | Total BCG n=1380 | Scar prevalence n/N (%) | Univariate OR (95% CI) | Multivariate OR (95% CI) |
|---|---|---|---|---|
| Sex | | | | |
| Male | 353 | 219 (62.0) | 1 (reference) | 1 (reference) |
| Female | 1027 | 784 (76.3) | 1.97 (1.52-2.56), p<0.001 | 2.04 (1.54-2.71), p<0.001 |
| Age | | | | |
| 18-49 | 912 | 701 (76.9) | 1 (reference) | 1 (reference) |
| ≥50 | 468 | 302 (64.5) | 0.55 (0.43-0.70), p<0.001 | 0.41 (0.31-0.54), p<0.001 |
| Nutritional status (BMI) | | | | |
| Normal weight (18.5-24.9) | 621 | 471 (75.8) | 1 (reference) | - |
| Underweight (<18.5) | 11 | 7 (63.6) | 0.56 (0.16-1.93), p=0.4 | |
| Pre-obesity (25.0-29.9) | 451 | 311 (69.0) | 0.71 (0.54-0.93), p=0.01 | |
| Obesity class I (30.0-34.9) | 163 | 114 (69.9) | 0.74 (0.51-1.09), p=0.1 | |
| Obesity class II (35.0-39.9) | 62 | 47 (75.8) | 1.00 (0.54-1.84), p=0.9 | |
| Obesity class III (>40) | 32 | 26 (81.3) | 1.38 (0.56-3.42), p=0.5 | |
| Unknown | 40 | 27 (67.5) | NA | |
| Smoker | | | | |
| No | 1298 | 948 (73.0) | 1 (reference) | - |
| Yes | 82 | 55 (67.1) | 0.75 (0.47-1.21), p=0.2 | |
| Diabetes | | | | |
| No | 1359 | 988 (72.7) | 1 (reference) | - |
| Yes | 21 | 15 (71.4) | 0.94 (0.36-2.44), p=0.9 | |
| Chronic respiratory disease | | | | |
| No | 1276 | 920 (72.1) | 1 (reference) | - |
| Yes | 104 | 83 (79.8) | 1.53 (0.93-2.51), p=0.1 | |
| Chronic cardiovascular disease | | | | |
| No | 1274 | 934 (73.3) | 1 (reference) | - |
| Yes | 106 | 69 (65.1) | 0.68 (0.45-1.03), p=0.07 | |
| BCG history | | | | |
| 1st BCG | 651 | 463 (71.1) | 1 (reference) | 1 (reference) |
| BCG revaccination | 729 | 540 (74.1) | 1.16 (0.92-1.47), p=0.2 | 1.58 (1.19-2.08), p=0.001 |
| Previous known LTBI | | | | |
| No | 1353 | 983 (72.7) | 1 (reference) | - |
| Yes | 15 | 9 (60.0) | 0.56 (0.20-1.60), p=0.3 | |
| Unknown | 12 | 11 (91.7) | NA | |
| Previous TST | | | | |

| | | | | |
|---|---|---|---|---|
| Negative/None | 1021 | 739 (72.4) | 1 (reference) | - |
| Positive (>5mm) | 115 | 88 (76.5) | 1.24 (0.79-1.96), p=0.3 | |
| Unknown | 244 | 176 (72.1) | NA | |
| BCG batch | | | | |
| 118006D | 591 | 431 (72.9) | 1.02 (0.80-1.30), p=0.9 | - |
| 118017F | 789 | 572 (72.5) | 0.98 (0.77-1.24), p=0.9 | |
| Co-administered influenza vaccine† | | | | |
| No | 192 | 152 (79.2) | 1 (reference) | 1 (reference) |
| Yes | 1188 | 851 (71.6) | 0.66 (0.46-0.96), p=0.03 | 0.57 (0.39-0.84), p<0.01 |
| Post-injection wheal* | | | | |
| Yes | 1231 | 899 (73.0) | 1 (reference) | 1 (reference) |
| No | 19 | 11 (57.9) | 0.51 (0.20-1.27), p=0.1 | 0.34 (0.13-0.90), p=0.03 |
| Unknown | 130 | 93 (71.5) | NA | - |
| Vaccinator experience | | | | |
| ≥20 vaccinees | 1199 | 864 (72.1) | 1 (reference) | - |
| 0-19 vaccinees | 181 | 139 (76.8) | 1.28 (0.89-1.85), p=0.2 | |
