# Supplementary material for: Factors influencing scar formation following Bacille Calmette-Guérin (BCG) vaccination
Source: Heliyon. 2023 Apr 26;9(6):e15821. doi: 10.1016/j.heliyon.2023.e15821 (PMC10360588; doi:10.1016/j.heliyon.2023.e15821)
Supplement: Multimedia component 6 [file mmc6.pdf]

Supplemental Table 2. Sensitivity analysis; factors investigated for association with BCG scar formation (Brazil)
Abbreviations: BCG, Bacille Calmette-Guérin; BMI, body mass index; LTBI, latent tuberculosis infection; NA, not applicable; OR, odds ratio; TST, tuberculin skin test
*Wheal response (yes/no) analysed for participants who received one BCG dose only.
†Variable omitted from the logistic regression model due to perfect prediction of a scar outcome.
Significant factors (p-value < 0.2) resulting from the univariate logistic regression analysis were included as possible covariates in a multivariate logistic regression model. The model presented in the table was created using backward stepwise exclusion of factors with p-value > 0.05, using sequential model testing.

Factor	Total BCG n=1222	Scar prevalence n/N (%)	Univariate OR (95% CI)	Multivariate OR (95% CI)
Sex				
Male	350	279 (79.7)	1 (reference)	1 (reference)
Female	872	753 (86.3)	1.61 (1.16-2.23), p<0.01	1.65 (1.18-2.30), p<0.01
Age				
18-49	977	856 (87.6)	1 (reference)	1 (reference)
≥50	245	176 (71.8)	0.36 (0.26-0.51), p<0.001	0.37 (0.26-0.52), p<0.001
Nutritional status (BMI)				
Normal weight (18.5-24.9)	387	327 (84.4)	1 (reference)	-
Underweight (<18.5)	18	15 (83.3)	0.59 (0.26-3.27), p=0.9	
Pre-obesity (25.0-29.9)	498	418 (83.9)	0.96 (0.67-1.38), p=0.8	
Obesity class I (30.0-34.9)	212	187 (88.2)	1.37 (0.83-2.26), p=0.2	
Obesity class II (35.0-39.9)	81	66 (81.5)	0.81 (0.43-1.51), p=0.5	
Obesity class III (>40)	21	14 (66.7)	0.37 (0.14-0.95), p=0.04	
Unknown	5	5 (100.0)	NA	
Smoker				
No	1090	916 (84.0)	1 (reference)	-
Yes	132	116 (87.9)	1.38 (0.80-2.38), p=0.3	
Diabetes				
No	1167	988 (84.7)	1 (reference)	-
Yes	55	44 (80.0)	0.72 (0.37-1.43), p=0.4	
Chronic respiratory disease				
No	1162	980 (84.3)	1 (reference)	-
Yes	60	52 (86.7)	1.21 (0.56-2.58), p=0.6	
Chronic cardiovascular disease				
No	1047	894 (85.4)	1 (reference)	-
Yes	175	138 (78.9)	0.64 (0.43-0.95), p=0.03	
BCG history				
1st BCG	40	31 (77.5)	1 (reference)	-
BCG revaccination	1182	1001 (84.7)	1.61 (0.75-3.43), p=0.2	
Previous known LTBI				
No	1218	1028 (84.4)	1 (reference)	-
Yes	1	1 (100.0)	NA†	
Unknown	3	3 (100.0)	NA	

Previous TST				
Negative/None	1150	970 (84.3)	1 (reference)	-
Positive (>5mm)	42	38 (90.5)	1.76 (0.62-5.00), p=0.3	
Unknown	30	24 (80.0)	NA	
BCG batch				
118019D	658	536 (81.5)	0.60 (0.44-0.83), p<0.01	0.63 (0.45-0.87), p<0.01
119039B	3	3 (100.0)	NA†	NA†
119053A	557	491 (88.2)	1.71 (1.23-2.35), p=0.001	1.58 (1.13-2.19), p<0.01
Unknown	4	2 (50.0)	NA	NA
Post-injection wheal*				
Yes	1212	1024 (84.4)	1 (reference)	
No	6	6 (100.0)	NA†	
Unknown	4	2 (50.0)	NA	
Vaccinator experience				
≥20 vaccinees	1098	926 (84.3)	1 (reference)	-
0-19 vaccinees	124	106 (85.5)	1.09 (0.65-1.85), p=0.7	
